# Behavioural and psychological symptoms in dementia and the challenges for family carers: systematic review

**DOI:** 10.1192/bjp.bp.114.153684

**Published:** 2016-05

**Authors:** Alexandra Feast, Martin Orrell, Georgina Charlesworth, Nina Melunsky, Fiona Poland, Esme Moniz-Cook

**Affiliations:** **Alexandra Feast**, MPhil, Division of Psychiatry, University College London, and Research and Development Department, North East London National Health Service (NHS) Foundation Trust, Ilford; **Martin Orrell**, PhD, Research and Development Department, North East London NHS Foundation Trust, Ilford, and Institute of Mental Health, University of Nottingham; **Georgina Charlesworth**, PhD, Research and Development Department, North East London NHS Foundation Trust, Ilford, and Research Department of Clinical, Educational, and Health Psychology, University College London; **Nina Melunsky**, MSc, Research and Development Department, North East London NHS Foundation Trust, Ilford; **Fiona Poland**, PhD, Faculty of Medicine and Health Sciences, University of East Anglia; **Esme Moniz-Cook**, PhD, Faculty of Health and Social Care, University of Hull, UK

## Abstract

**Background**

Tailored psychosocial interventions can help families to manage behavioural and psychological symptoms in dementia (BPSD), but carer responses to their relative's behaviours contribute to the success of support programmes.

**Aims**

To understand why some family carers have difficulty in dealing with BPSD, in order to improve the quality of personalised care that is offered.

**Method**

A systematic review and meta-ethnographic synthesis was conducted of high-quality quantitative and qualitative studies between 1980 and 2012.

**Results**

We identified 25 high-quality studies and two main reasons for behaviours being reported as challenging by family carers: changes in communication and relationships, resulting in ‘feeling bereft’; and perceptions of transgressions against social norms associated with ‘misunderstandings about behaviour’ in the relative with dementia. The underlying belief that their relative had lost, or would inevitably lose, their identity to dementia was a fundamental reason why family carers experienced behaviour as challenging.

**Conclusions**

Family carers' perceptions of BPSD as challenging are associated with a sense of a declining relationship, transgressions against social norms and underlying beliefs that people with dementia inevitably lose their ‘personhood’. Interventions for the management of challenging behaviour in family settings should acknowledge unmet psychological need in family carers.

Behavioural and psychological symptoms of dementia (BPSD) such as agitation, aggression, calling out repeatedly, sleep-disturbance, wandering and apathy affect up to 90% of people with dementia, and are associated with poor outcomes such as distress in both the person with dementia and the carer, long-term hospital stay, misuse of medication and increased healthcare costs. Behaviour in dementia care can be described as ‘challenging’ when it causes distress to the person or others (such as the family carer), thus threatening the quality of life of one or both parties. The relationship between BPSD and quality of life varies from person to person, and common or frequent BPSD are not necessarily the most challenging for family carers.^1^ Moreover, the carer's own characteristics, independent of dementia severity or other patient factors, can contribute to the development of aggressiveness.^2^ Carer responses to BPSD vary, and how carers accept their situation and manage dementia-related problems can influence the course of BPSD.^3^ This may be why, even when families receive professional support, two-thirds indicate an unmet need associated with how to deal with BPSD.^4^ A recent Cochrane review concerning the management of challenging behaviour in dementia noted that all eleven studies of effective interventions with family carers involved various psychotherapeutic or counselling approaches directed at the carer.^5^ However, the nature and range of carer needs that are addressed within these individually tailored counselling interventions are not clear, thus making the type of support less easy to apply widely in the management of BPSD in family settings. Little is known about the particular psychosocial and emotional needs of family carers in dealing with BPSD. We reviewed the literature in order to understand why the impact of BPSD varies from carer to carer, and to consider how personalised interventions for family carers experiencing challenging behaviour in dementia could be improved.

## Method

We systematically reviewed studies that used both qualitative and quantitative methods to describe family carer experiences and reports of BPSD. The meta-ethnographic approach was used to extract the richest possible data-set,^6^ since this widely used method of synthesising closely related qualitative data has also been used with quantitative data.^7^ The method is interpretative in nature, focusing on drawing out analogies and relationships between concepts and metaphors that may be hidden within individual studies, and translating these into a meta-ethnographical synthesis.^6^ Meta-ethnography allows researchers to go beyond the purely summative findings associated with traditional narrative reviews. This method suited our particular research question, which was to have a better understanding of the nature of challenging behaviour in dementia in family settings; that is, to ascertain why some family carers might appraise behaviour as challenging, whereas others have less difficulty in coping.

### Selection of studies

A search strategy combining medical subject headings and text words relating to dementia, BPSD, mental disorders and behaviours, behaviours, elder care, significant others, carers, family, daughters, aged, carer burden, carer strain, and cost of illness was devised and adapted for five electronic databases (see online supplement DS1). Studies were restricted to those in the English language published between 1980 and April 2012, and to those that had a family carer's account of BPSD and/or the reasons why they felt these BPSD were challenging. Spousal and non-spousal carers were included. Reviews, conference proceedings, conference abstracts, theses, supplements, reports, letters and non-peer-reviewed articles were excluded.

### Quality appraisal

Three reviewers (A.F. and two assistants) assessed relevant papers for methodological quality; disagreements were resolved by discussion. The Critical Appraisal Skills Programme (CASP) checklist was used for qualitative and mixed-methods studies and the 27-item Downs & Black checklist for quantitative studies.^8,9^ Quality thresholds for high-quality studies were those used in previous systematic reviews:^10,11^ 70% or more on the CASP or 75% or more on the Downs & Black checklist. Agreement was measured using Cohen's κ weighted for closeness of scores.

### Data extraction and meta-ethnographic synthesis

To determine why BPSD are appraised as challenging by families, the meta-ethnographic procedure described in supplement DS2 was followed.^6^

## Results

**Fig. 1:**
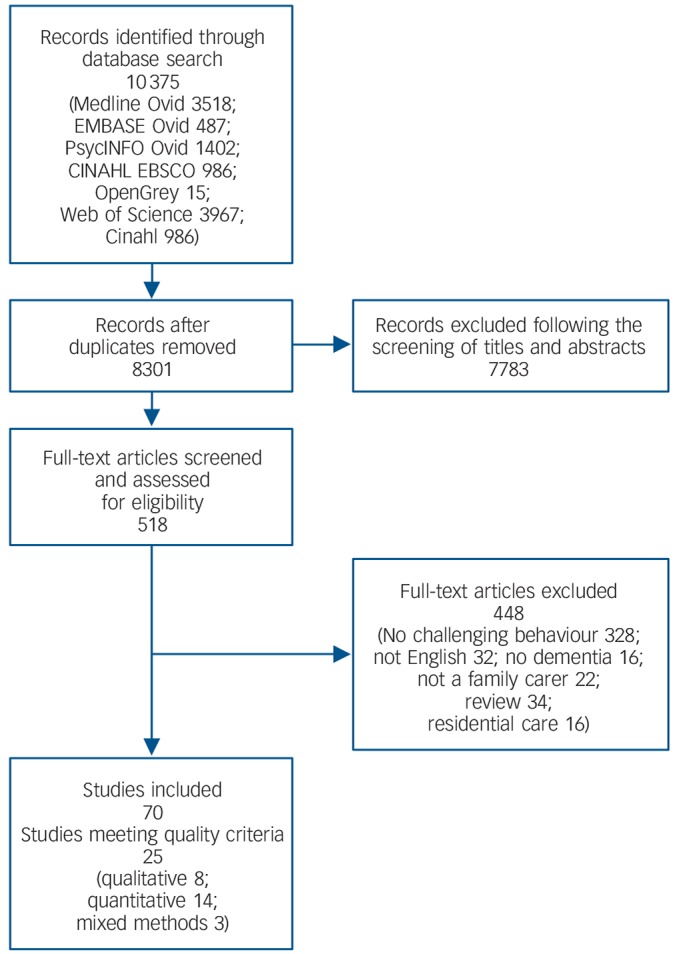
Study selection process.

A total of 10 375 references were identified (Fig. 1); of the 70 studies that met the inclusion criteria prior to quality assessment, 45 were quantitative, 18 were qualitative and 7 were mixed methods. Twenty-five studies were graded as high quality, 39 as moderate quality (5–7 on the CASP; 50–75% on the Downs & Black checklist) and 6 as poor quality (<5 on the CASP; <50% on the Downs & Black checklist). Characteristics of the included studies are shown in online Table DS1. Two studies exclusively focused on participants with young-onset dementia (i.e. onset before age 65 years).^12,13^

### Quality assessment of included studies

Levels of agreement between the three independent reviewers ranged from moderate (κ = 0.52, 95% CI 0.07–0.97) to substantial (κ = 0.78, 95% CI 0.37–1.00).^14^ Lower agreement was noted for items on the qualitative appraisal CASP checklist which was also used for mixed-method studies (*n* = 11 studies).

### Third-order constructs: synthesis

Our inclusion criteria allowed extraction of data on both qualitative and quantitative accounts of BPSD in family care settings, but some studies relied totally on a structured interview, such as the Neuropsychiatric Inventory,^15^ in reporting the accounts of family carers. Therefore, six studies could not contribute to the themes that emerged since neither participant-derived (first-order) nor author-derived (second-order) data offered an explanation for why behaviour was appraised as challenging.^16–21^ Two studies offered no data in the form of first-order constructs but did provide second-order data which supported our theme categories.^22,23^

We identified two third-order constructs that contributed to the understanding of why behaviours are appraised as challenging. The first, ‘feeling bereft’, included theme categories which in some way conditioned communication and the relationship. Themes associated with the second construct in some way reflected behaviours that were appraised as a transgression of social norms, where a carer failed to understand why the person with dementia behaved in certain ways. These provided a foundation for ‘misunderstandings about behaviour’ and carer interpretations of the meaning of their relative's communications (see Appendix). Conceptual groups identified from first- and second-order constructs were all supported by at least one high-quality study. Each theme was supported by data extracted from both qualitative and quantitative studies.

### Feeling bereft

A strong sense of ‘feeling bereft’ was associated with changes in communication and the impact of BPSD on the relationship.

#### Changes in communication

Withdrawal behaviours described as ‘apathy’^16–19,24–28^ in studies that used (for example) the Neuropsychiatric Inventory were commonly distressing for families,^18,19,25,27^ where items such as ‘shows seldom or no interest in news about friends and relatives’ were endorsed.^18^ As lack of interest was noticed,^17,22,26,27^ the range of shared pleasurable activities also declined:

‘She'd watch a hockey game with me and all of a sudden it seems to be gone and there's no interest’ (p. 82).^26^

Positive communication between people with dementia and their carers was also undermined by repetitive interactions, such as when repeated questioning resulted in the need for providing repeated information.^29–32^ The sense of declining conversation, ‘You can't have a discussion any more, not a real discussion’ (p. 81),^26^ exacerbated the distress associated with challenging behaviour in dementia:

‘Not being able to talk with your wife or communicate in any way is the most distressing part of it’ (p. 230).^33^

The contribution of BPSD to deteriorating communication with the relative, ‘It is awful to be around a man all day who doesn't communicate. I would rather talk to someone about unimportant or stupid things, than not talk at all’ (p. 153),^27^ combined with a developing sense of isolation,^26^ ‘You can somehow communicate with a dead person in a grave, and that's better than visiting a living dead person when there are no signs whatsoever of communication’ (p. 230),^33^was distressing, difficult to cope with and therefore appraised as ‘challenging’ by family carers.^24,26,27,30,31,33^ The extracted first- and second-order constructs for this theme category can be seen in online Table DS2.

#### Changes in relationships

As conversation and shared activities declined there was a detrimental effect on the relationship, since the carer missed companionship,^34^ ‘I miss my mother … It is difficult to explain how much one can miss someone’ (p. 687),^34^ and reciprocal interactions, ‘You can try everything, but you get no response at all. That is very difficult for me’ (p. 153).^27^ The ongoing struggle to live with a changed relationship left some with a sense of rejection:^12,26,27,34,35^

‘I've always been able to trust my mother to support me one hundred per cent, but little by little all her support has vanished. Before I could phone her and ask her things or tell her about everything that was happening and I always got an honest answer. I could phone and complain and things like that’ (p. 230).^33^

Others no longer felt loved:

‘I miss the love between us. That is the most important problem. I miss his arm around me when I am cooking dinner’ (p. 153).^27^

This sense of loss, or ‘slipping away’,^12^ of the person they once knew became acute and in some cases resulted in grief,^34^ particularly when carers appraised their loved one as ‘not knowing or recognising them.’^12,27,33,34,35^ The changed role and new responsibilities, such as role reversal from provider to dependant,^12,33,34^ and from that of protection and nurturing to having to provide care:^34^ ‘I don't want to be a mother for my mother …’ (p. 687),^34^ engendered for some a sense of role captivity^30^ and strain.^12,13,26,34–36^

The impact of BPSD on the relationship between the person with dementia and some family carers was the loss of the emotional bond of love^27,34,35^ and nurture resulting in isolation,^26,27,34^ feelings of rejection,^27^ and even grief.^12,34^

### Misunderstandings about behaviour

The theme of misunderstandings about behaviour reflected the perception that the relative had transgressed social norms and was associated with the carer's personal or sociocultural expectations.

#### Personal expectations

Although families were aware that their relative had dementia, some had difficulty in understanding the ‘meaning’ of their relative's communicated behaviour, particularly when this was appraised as ‘out of character’,^12,34^ or within their relative's control.^22^ Thus repeated questioning about ‘forgotten’ tasks or activities that might be worrying the person with dementia,^29,31,32^ requiring the carer to constantly repeat requests to no end, becomes challenging:

‘Well, I think it's very stressful and it's the repeating, repeating and repeating, and you expect him to remember something and then he doesn't … it is stressful’ (p. 221).^31^

Accusations of stealing made by a person with dementia who has misplaced valued items become troublesome when interpreted as personally offensive,^19,25,37^ and when frustration in a relative with dementia is interpreted as aggression, the behaviour becomes stressful. This undermines the carer's efficacy in coping with BPSD, and the relative's behaviour is therefore appraised as challenging,^12,27,19–22,25,27,28,30,34–37^ since the carer feels unable to respond effectively: ‘She thinks I have met someone else. It isn't true and I don't know how to handle it’ (p. 687);^34^ consequently the caregiving situation can deteriorate. This may also be the case with newly occurring behaviours as the carer may not yet know or feel confident in how to respond effectively.^23^

Understanding helped some carers, but levels of understanding and thresholds of carer tolerance of their relatives' communications of distress or discomfort can vary,^12,34^ even within families:

‘ … try to explain them [behaviours] to my mum. She does understand them but it is hard for her to keep them in mind’ (p. 465).^12^

The personal expectations of carers, such as the level of care they wish to provide, and their own need for care due to advanced age and disabilities, can also affect thresholds of tolerance of the relative's behaviour, and the efficacy of the carer to respond effectively to BPSD.^25,28,31,38^

#### Sociocultural expectations

Gender and sociocultural expectations can trigger embarrassment and shame for the carer,^35,36^ when BPSD such as disinhibition transgress expectations in public.^12,13,28^ Although carers can use strategies to manage BPSD, strongly held expectations can also lead to distress and perceptions of challenging behaviour:

‘He did go to the toilet in the bed and I didn't know what to do the first time … I don't wanna clean it … I'm Italian … my mum still leaves my clothes on the bed in the morning … how am I supposed to clean an 80-year-old … I did and then I went outside and spewed my guts out’ (p. 293).^35^

The sometimes ‘expected submissive role’ of female carers in family subsystems can also undermine the ability of a carer to use strategies that require the provision of clear, firm instructions to assist the person with dementia.^36^

### Line of argument synthesis

The sense that people with dementia inevitably lose their identity to the disorder was a key explanatory theme for challenging behaviour in family settings. Carer perceptions were anticipated: ‘Ummm, well if it gets worse and like he can't remember who me or my sister or anybody is then that would be hard to … not very nice’ (p. 473),^12^ or experienced: ‘I have come to terms with the fact that my dad is not my dad any more’ (p. 467),^12^ leading to a deconstruction of the person's ‘lived life’ associated with metaphors of decline to childhood,^26,30,34^ ‘My mother is like a child’ (p. 687),^34^ or worse.^37^ Thus the common social construction about dementia – that is, the belief that the person would inevitably become ‘no longer human’ – can be seen in family carers who have difficulty in coping with BPSD:

‘It's an awful illness, because Mom no longer exists even though she's still there’ (p. 230).^33^

In accordance with this fear of dementia, the proposition of becoming dehumanised – of losing one's identity – is the construction that dementia is an invader which creeps up on people and steals them from themselves, resulting in metaphoric comparisons of people with dementia as the ‘living dead’.^39^

## Discussion

We identified key constructs for the understanding and management of challenging behaviour in family care settings: namely, issues relating to changes in communication, companionship or reciprocity in relationships, and/or carer perceptions of transgressions against social norms. These are consistent with commentaries in which interpersonal, family and social contexts are noted as contributory factors in the development and course of BPSD.^5,26^ The identified themes from this meta-ethnography provide a psychological understanding of unmet need in family carers who struggle to accept and adjust to their changed circumstances.^35,36,40^ For these carers emotions associated with loss of the relationship and perceptions of antisocial behaviour can act as barriers to their effective responses to BPSD. Underlying the experience of challenging behaviour were beliefs that the person with dementia would inevitably lose his or her identity to the disorder. Therefore for some family carers, metaphors of dementia as the ‘living dead’ appeared salient in undermining their effective responses to BPSD.

### Unmet need in family carers

Difficulties arise for the carer when a behaviour that is appraised as being out of character for the relative is overgeneralised to indicate a complete change in the person, e.g. ‘All were mourning over the loved one who was no longer the person they had known’ (p. 448).^40^ Our findings suggest that underlying these perceptions is the carer's subtle but sustained distress resulting from loss of the emotional bond with the relative with dementia, and the carer's consequent own unmet need for nurture, care and emotional security.

Studies of attachment theory in dementia care describe why some carers adjust effectively to the changed circumstances associated with dementia and thus experience reduced challenging behaviour, whereas others do not.^41^ For example, Ingebretsen & Solem noted that spouse carers' ability to cope with loss and changes was dependent on their own needs for emotional safety: secure attachments enabled adjustment, whereas those who did not cope well were seen as in need of high levels of support, empathy and individual care.^42^ Therefore, for carers who have difficulty in coping with BPSD, initial emotional support may enhance therapeutic engagement and their readiness to use BPSD management strategies.^43^ Carers who cope with BPSD through ‘emotional distancing’,^44^ which can result in ‘deconstruction’ of the person with dementia, may then be unable to provide the support that is required by their relative,^44^ including strategies to respond effectively to dementia-related problems. Examples of emotional distancing include the shift from an ‘us’ identity for spouses to a sense of ‘me and them’,^45^ which at its worst reinforces fear and stigmatised beliefs,^39^ such as descriptions of the ‘living dead’ where the person with dementia is entirely ‘lost’ to the carer.^33^

Misunderstandings about the meaning of behaviours when these are perceived as transgressing interpersonal social norms and expectations can precipitate strong emotions that affect the carer's responses. For example, feelings of embarrassment and shame become barriers to help-seeking,^46^ explaining why families feel ill-equipped in dealing with BPSD.^4^ Although there are similarities in the experiences and perceptions of culturally and linguistically diverse family carers, there are also differences across groups.^35^ Therefore, interventions that take account of social and cultural factors that may contribute to variation in carer adjustment to changing circumstances,^46^ and their approaches to BPSD,^3^ can be helpful in bolstering some caregiving contexts.

### Understanding and intervening

Consistent with the literature,^47^ our first explanatory construct for variation in carer responses to BPSD found noteworthy accounts of changes in communication due to dementia which appeared to reduce the quality of dyadic interactions, with a consequent declining relationship and a strong sense of loneliness: this we describe as the carer ‘feeling bereft’. Interventions to address the carer's need for understanding and managing changes in communication are at an early stage of development,^48^ but one preliminary investigation reported reductions in BPSD and positive outcomes for the caring experience.^48^ However, communication interventions are untested in caregiving contexts where there are known risk factors to carer adjustment to the changed circumstances associated with dementia,^49^ or where role captivity may undermine meaning in the caregiving context.^30,50^

Multicomponent interventions in family care settings have a developing evidence base for the management of challenging behaviour in dementia.^16^ For example, one component of the intervention set out in the Seattle carer training protocols (STAR-C) focused on ‘increasing pleasant events’.^51^ This has the potential to help adjustment to the changed circumstances and thus moderate the sense of ‘feeling bereft’ (see Appendix). An adaptation of this multicomponent programme to a UK setting also reduced BPSD and carer responses to behaviour, and improved carer mood.^52^ More recently, the Strategies for Relatives (START) psychoeducation programme,^53^ which included an ‘understanding behaviour’ component, had a positive impact on family carer mood. This programme used cognitive–behavioural techniques to help carers identify unhelpful appraisals and identify more adaptive alternatives. Our review adds to this literature by suggesting that interventions should additionally target the carer's unmet need within each caregiving context by employing strategies to address the carer's underlying assumptions and core beliefs.

### Strengths and limitations

Our study is the first, as far as we know, to review variation in family carer responses to BPSD. We used rigorous methods for systematic review, including a comprehensive search of five electronic databases and a selection of high-quality studies against predetermined criteria. Our review was inclusive in that it accessed both quantitative and qualitative studies which were heterogeneous in methodological design, relationship type, onset of dementia and country setting. There is a risk with meta-ethnography of compromising the integrity of the individual studies, but we made extra efforts to retain context and holism for the studies reviewed during each stage of the synthesis. Two experienced clinicians (G.C. and E.M.-C.) examined extracted data to ensure context and meaning from the original data (first-order constructs) were not lost during the synthesis. One disadvantage of our decision to include only high-quality studies was a loss of accounts from subthreshold studies, particularly qualitative and mixed-methods studies. To counter this we checked our findings against data from subthreshold studies to ensure completeness. Although additional theme categories from these studies were identified, they were in accordance with the data that shaped the current third-order constructs and subsequent line of argument. Caution is needed over the potential over-influence of two studies of caring experience in young-onset dementia.^12,13^ Our third-order construct ‘misunderstandings about behaviour’ was underpinned by perceived transgressions of social norms and qualitative accounts for ‘personal expectations’ (see Appendix), which drew heavily on one of these two studies. For this theme generalisation cannot be assumed, since disturbances in social behaviour are often a distinct feature of a dementia subtype that was present in some participants.^12^ A limitation to our findings was that six – almost a quarter – of the included studies did not offer data for synthesis on why carers might be distressed by BPSD. These studies reported quantitative or mixed-methods data.^16–21^ The information from these studies was weighted towards the structure of the questionnaire, for example the Neuropsychiatric Inventory, so it was not possible to extract first- or second-order constructs. Nevertheless, the synthesis has provided novel insight into how support programmes for family carers can be improved by understanding why some BPSD are experienced as challenging.

### Implications for research and practice

The Cochrane review of an intervention for challenging behaviour noted that most successful programmes had elements of support for the family carer as well as addressing unmet need in the care recipient.^5^ Interventions that focus on carer knowledge aim to reduce unrealistic expectations, increase feelings of competency, increase positive comparisons and thus reduce the number of behaviours that are perceived as challenging. However, our review suggests that attention to the wider caregiving context such as the carer's unmet psychological needs and negative feelings – feeling bereft and/or ashamed – are also important interventions for the management of challenging behaviour. It is possible that carers who can recognise the role of the illness in the loss of identity, and retain the conceptualisation of their relative with dementia as the person they have always known and loved, will continue to have a fulfilling relationship. Consequently, the companionship and feelings of care and nurture gained from the relationship will help to reduce carers' perception of behaviours as challenging, thus improving their ability to cope. Future interventions could also investigate the effect of helping family carers to redefine their new role in ways they find acceptable, identify new means of communicating with their relative, improve reciprocity with the dyadic relationship, and create more opportunities for shared activities. In order to support families these interventions should be studied early on in the illness, since aspects of our conceptualisation of the caregiving context can be understood independently of dementia severity or other patient characteristics.

## Appendix

### Third-order constructs

Third-order constructs are listed with associated theme categories and themes for why behaviours are seen as challenging by family carers.

Feeling ‘bereft’

Changes in communication^12,26,27,29–33,34^

- repetitive interactions^29–32^
- decline in conversation and isolation^12,26,27,33,34^

Changes in relationships^12,13,26,27,33,35,36^

- loss of care and nurture – ‘feeling unloved/rejected’^27,33,35^
- loss of companionship^26,27,34^
- role reversal/captivity^12,13,33,34,36^

‘Misunderstandings about behaviour’ due to perceived transgressions against social norms

Personal expectations^12,22,23,25,28,31,35,34–38^

- understanding meaning and carer efficacy^12,22,23,34,37^
- carer threshold/tolerance^25,28,31,38^

Sociocultural expectations

- social context, gender and culture^28,35,36^
